# Reduced numbers of regulatory T cells in chronic heart failure seems not to be restored by cardiac resynchronization therapy

**DOI:** 10.1186/s12872-023-03109-x

**Published:** 2023-02-15

**Authors:** Sílvia Martins, Natália António, Tiago Carvalheiro, Paula Laranjeira, Ricardo Rodrigues, Lino Gonçalves, Cândida Tomaz, Artur Paiva

**Affiliations:** 1 Health Sciences Research Centre, University of Beira Interior (CICS-UBI), 6200-506 Covilhã, Portugal; 2 Ciências Biomédicas Laboratoriais, Instituto Politécnico de Castelo Branco, ESALD-Dr, Lopes Dias Health School, Castelo Branco, Portugal; 3 Department of Clinical Pathology, Unidade Local de Saúde de Castelo Branco, 6000-085 Castelo Branco, Portugal; 4 University of Coimbra, Coimbra Institute for Clinical and Biomedical Research (iCBR), University of Coimbra, Coimbra, Portugal; 5 Cardiology Department, Centro Hospitalar e Universitário de Coimbra, Coimbra, Portugal; 6 Institute of Pharmacology and Experimental Therapeutics/iCBR, Faculty of Medicine, University of Coimbra, Coimbra, Portugal; 7 Centro do Sangue e da Transplantação de Coimbra, Instituto Português do Sangue e da Transplantação, Coimbra, Portugal; 8 Flow Cytometry Unit, Department of Clinical Pathology, Centro Hospitalar e Universitário de Coimbra, Coimbra, Portugal; 9 Ciências Biomédicas Laboratoriais, Instituto Politécnico de Coimbra, ESTESC-Coimbra Health School, Coimbra, Portugal; 10 Chemistry Department, University of Beira Interior, Covilhã, Portugal; 11 Flow Cytometry Unit, Department of Clinical Pathology, Centro Hospitalar e Universitário de Coimbra, Praceta Mota Pinto, 3000-075 Coimbra, Portugal

**Keywords:** Chronic heart failure, Cardiac resynchronization Therapy, Immune response, T cells, cytokines profile

## Abstract

**Background:**

T cells have been implicated in the development and progression of inflammatory processes in chronic heart failure (CHF). Cardiac resynchronization therapy (CRT) has beneficial effects on symptoms and cardiac remodeling in CHF. However, its impact on the inflammatory immune response remains controversial. We aimed to study the impact of CRT on T cells in heart failure (HF) patients.

**Methods:**

Thirty-nine HF patients were evaluated before CRT (T0) and six months later (T6). Quantification of T cells, their subsets, and their functional characterization, after in vitro stimulation, were evaluated by flow cytometry.

**Results:**

T regulatory (Treg) cells were decreased in CHF patients (healthy group (HG): 1.08 ± 0.50 *versus* (heart failure patients (HFP)-T0: 0.69 ± 0.40, *P* = 0.022) and remaining diminished after CRT (HFP-T6: 0.61 ± 0.29, *P* = 0.003). Responders (R) to CRT presented a higher frequency of T cytotoxic (Tc) cells producing IL-2 at T0 compared with non-responders (NR) (R: 36.52 ± 12.55 *versus* NR: 24.71 ± 11.66, *P* = 0.006). After CRT, HF patients presented a higher percentage of Tc cells expressing TNF-α and IFN-γ (HG: 44.50 ± 16.62 *versus* R: 61.47 ± 20.54, *P* = 0.014; and HG: 40.62 ± 15.36 *versus* R: 52.39 ± 18.66, *P* = 0.049, respectively).

**Conclusion:**

The dynamic of different functional T cell subpopulations is significantly altered in CHF, which results in an exacerbated pro-inflammatory response. Even after CRT, it seems that the inflammatory condition underlying CHF continues to evolve with the progression of the disease. This could be due, at least in part, to the inability to restore Treg cells levels.

*Trial registration*: Observational and prospective study with no trial registration.

**Supplementary Information:**

The online version contains supplementary material available at 10.1186/s12872-023-03109-x.

## Introduction

Chronic heart failure (CHF) is a common and debilitating disorder [1, 2] with significant rates of morbidity and mortality in modern societies [3]. The pathophysiology of CHF implicates progressive myocardial dysfunction associated with continuous ventricular remodeling, which is, by itself, a complex and multifactorial process [4, 5]. It is well established that multiple factors such as neurohumoral mediators, enzymes, oxidative stress and mechanical stress, as well as inflammation are involved in pathological left ventricular (LV) remodeling and systolic dysfunction [4].

Multiple studies have demonstrated that the pro-inflammatory response contributes to the pathophysiology of CHF and that its up-regulation implies a dismal prognosis in affected patients [6–8].

T helper (Th) cells play a key role in several chronic inflammatory disorders and numerous studies propose their active participation in the pathogenesis of CHF [6–8]. The proinflammatory Th1 and Th17 cells are increased in CHF patients [9, 10]. Conversely, current evidence suggests that down-regulation or insufficient recruitment of regulatory T (Treg) cells results in worsened ventricular remodeling [11–13]. Moreover, it has been described that the Th1/Th2 imbalance [14] and the polarization of type 1 Th cells play a pathogenic role in CHF [10].

Treg cells seem to be reduced in different heart failure (HF) aetiologies and associated with a dismal prognosis. They can also participate in the pathophysiology of CHF by assuming an antiangiogenic and profibrotic profile Th1 like cells [15].

T cytotoxic (Tc) cells and their role in CHF has received less attention however, they seem to contribute to immune-mediated damage. Recent studies suggest that Tc cells contribute to cardiomyocyte apoptosis, adverse ventricular remodeling and deterioration of myocardial function [16]. Moreover, abundant Tc lymphocytes producing large amounts of IFN-γ were found in ischemic failing hearts [11].

Cardiac resynchronization therapy (CRT) is an effective treatment for patients with severe CHF. Based on biventricular pacing, CRT restores the electromechanical desynchrony of the heart, improving LV systolic function and reducing patient symptoms, re-hospitalizations and mortality [4, 17, 18]. Limited evidence suggests that CRT can reduce inflammatory mediators in HF patients [19], but the association between CRT response, T cells and cardiac remodeling in CHF is far from being understood [4]. We performed an exploratory study to generate hypotheses based on the impact of CRT on the frequency and functional activity of T cells subpopulations in CHF patients submitted to CRT, by comparing baseline with post-CRT data.

## Methods

### Patient population

A total of thirty-nine consecutive and ambulatory patients with advanced heart failure, scheduled for CRT, were prospectively included in this study between 2010 and 2013; their mean age was 61.4 ± 10.5 years, 26 patients were male and 13 were female (Table 1). Patients were assisted and followed-up in a tertiary Cardiology Department (Centro Hospitalar e Universitário de Coimbra). To calculate the sample size, the software G*Power 3.1 was used [20]. Prior analysis was performed determining that 35 subjects would be needed for the study (Effect size dz:0.7, α error probability:0.05, power:0.80). Additionally, four elements were added to the sample as a matter of convenience.

**Table 1:** Clinical characterization of responders and non-responders to CRT

	Global Population Mean ± standard deviation (n = 39)	Responders Mean ± standard deviation (n = 21)	Non-Responders Mean ± standard deviation (n = 18)	*P* value *Responders vs Non-responders*
Baseline assessment				
Gender (Male/Female)	26 / 13	14 / 7	12 / 6	1
Aetiology (Non-Ischemic/Ischemic)	29 / 10	17 / 4	12 / 6	0.418
NYHA (II/III/IV)	8/ 27/ 4	4/ 16/ 1	4/ 11 /3	0.465
LV lead position (L/PL/A/AL)	17/11/3/8	12/4/2/3	5/7/1/5	0.228
Age (years)	61.4 ± 10.5	**65.2 ± 9.6**	**56.9 ± 9.8**	**0.015**
LVEF (%)	24.9 ± 6.9	23.8 ± 6.5	26.3 ± 7.4	0.309
LVESV (mL)	190.2 ± 84.9	180.1 ± 55.3	202.1 ± 111.0	0.903
LVEDV (mL)	244.2 ± 83.9	233.3 ± 58.9	257.1 ± 106.8	0.583
QRS	148.4 ± 30.6	144.5 ± 22.1	151.4 ± 36.6	0.866
Total Leukocytes (× 10^3^ µl)	8.4 ± 1.7	8.1 ± 1.6	8.7 ± 1.8	0.242
hsCRP (mg/L)	5.6 ± 6.0	5.2 ± 5.1	5.9 ± 6.9	0.934
BNP (pg/mL)	362.8 ± 358.7	264.3 ± 214.8	461.3 ± 448.1	0.362
Uric Acid (mg/dL)	6.0 ± 1.7	5.6 ± 1.5	6.5 ± 1.9	0.231
Cholesterol (mg/dL)	185.9 ± 56.5	194.47 ± 61.85	175,81 ± 49.55	0.466
HDL Cholesterol (mg/dL)	44.4 ± 11.7	**48.53 ± 11.04**	**39,56 ± 10.84**	**0.031**
LDL Cholesterol (mg/dL)	117.4 ± 46.3	121.47 ± 49.98	111,41 ± 42.33	0.499
Triglycerides (mg/dL)	132.8 ± 55.2	122.37 ± 50.20	146.00 ± 59.68	0.252
After CRT				
Total Leukocytes (× 10^3^ µl)	8.3 ± 1.8	8.1 ± 1.5	8.6 ± 2.1	0.220
LVEF (%)	33.9 ± 10.8	**39.1 ± 9.8**	**27.6 ± 8.4**	**0.001**
LVESV (mL)	151.4 ± 96.0	**100.4 ± 36.7**	**215.1 ± 109.6**	** < 0.001**
LVEDV (mL)	220.2 ± 108.5	**168.9 ± 50.3**	**284.4 ± 127.9**	**0.001**
hsCRP (mg/L)	4.1 ± 4.6	2.6 ± 1.8	6.2 ± 6.4	0.288
BNP (pg/mL)	245.3 ± 334.6	**139.6 ± 164.1**	**403.9 ± 456.0**	**0.043**

Bold: Statistically significant differences (*P* < 0.05)

*NYHA* New York Heart Association, *L* Lateral, *PL* posterolateral, *A* anterior, *AL* anterolateral, *LVEF* left ventricular ejection fraction, *LVESV* left ventricular end-systolic volume, *LVEDV* left ventricular end-diastolic volume, *hsCRP* high sensitivity C-reactive protein, *BNP* B-type natriuretic peptide, *HDL Cholesterol* high-density lipoprotein cholesterol, *LDL Cholesterol* low-density lipoprotein cholesterol

At the time of inclusion, patients were under stable, optimal pharmacological therapy for CHF, including an angiotensin-converting enzyme (ACE) inhibitor or angiotensin receptor blocker, β-blocker, and aldosterone antagonist, unless contra-indicated or not tolerated. Inclusion criteria were restricted to patients with advanced heart failure, who met the criteria described in the guidelines, at that time, with class I recommendation for CRT: belonging to class II, III or IV according to NYHA (New York Heart Association); presenting LV ejection fraction (LVEF) ≤ 35%; QRS ≥ 120 ms with left bundle branch block morphology; and normal sinus rhythm. [21, 22]

Exclusion criteria were defined, in an attempt to exclude any changes in patients that could interfere with our assessment of the immune response and bias the results.

As exclusion criteria we included several conditions that might influence the inflammatory response such as clinical or biochemical manifestation of the presence of concomitant inflammatory disease [23]; active infections (which could trigger an inflammatory immune response); autoimmune or malignant diseases (since T cells perform an important role in immune response in autoimmune diseases and cancer [24, 25]); severe valvular disease or congenital heart disease (because evidence indicates that an inflammatory state and immune alterations are present in patients with valvular disease and congenital heart disease [26, 27]); cardiogenic shock (since implantation of CRT is not recommended at this stage); deep vein thrombosis or pulmonary embolism (because inflammation and coagulation are closely related, particularly in these diseases [28]); severe peripheral arterial occlusive disease (in which platelet activation and inflammation are usually abnormal [29]); severe and noncontrolled arterial hypertension (systolic blood pressure > 180 mmHg or diastolic > 110 mmHg) (it is described that the immune system, inflammation and hypertension are strongly related, and effector T and Treg cells play an important role in blood vessel constriction in hypertension [30]); recent trauma or surgery (< 1 month) (trauma can evoke a systemic reaction including a non-specific immune response which can result in multiple organ damage due to aggravated inflammation [31, 32]); recent major bleeding (< 6 months) requiring blood transfusion (considering that every blood component can promote inflammation [33]); renal insufficiency (creatinine > 2.0 mg/dl) (because inflammation is common in patients with chronic kidney disease) [34]); anaemia (haemoglobin < 8.5 g/dl) or thrombocytopenia (< 100,000/L) (since alterations in haematological parameters may be linked to inflammatory processes described in infections, sepsis and anaemia of inflammation [35, 36]); pregnancy (considering that inflammation is essential for female reproduction and pregnancy is itself an inflammatory state [37] and radiation is also contraindicated during pregnancy); atrial fibrillation (because this arrhythmia is often associated with enhanced inflammatory response, which seems to be implicated in the pathophysiology of atrial fibrillation); prior arterial coronary bypass surgery (an inflammatory reaction occurs after arterial coronary bypass surgery and contributes to postoperative organ dysfunction and coagulation disorders [38]); acute coronary syndrome, or percutaneous coronary intervention within three months (since dysfunctional immune response and inflammation also have been implicated in the pathogenesis of acute coronary syndrome [39]); previously implanted electronic cardiac devices (in order to contribute to the study homogeneity, we only included patients with a class I recommendation for CRT); and comorbidities associated with a life expectancy less than one year (given the severe comorbid condition of these patients, they could exhibit some degree of inflammation).

Patients taking medication that could interfere with immune response were also excluded. Patients taking regular nonsteroidal anti-inflammatory drugs or patients on anticoagulants, or those on continuous or intermittent intravenous inotropic therapy and excessive alcohol consumption or illicit drug abuse.

Candidate eligibility was ensured by baseline assessment of heart failure (HF) patients scheduled for CRT (T0) before the implantation of device. Patients were followed-up and re-evaluated for the same variables at six months after CRT (T6).

### Echocardiographic evaluation

Each patient underwent echocardiographic assessment at T0 and T6. Standard echocardiography was performed using a Vivid 7 (GE Healthcare, Oslo, Norway) and 1.7/3.4-MHz tissue harmonic transducer. Loops and three cardiac cycles were stored digitally and analysed offline using a customized software package (EchoPAC, GE Healthcare). The LV end-diastolic volume (LVEDV), LV end-systolic volume (LVESV) and LVEF were assessed by the biplane Simpson equation in apical four-chamber and two-chamber views [40, 41].

### Definition of response to CRT

We classified responders to CRT as patients who remained alive and showed at least a 15% reduction in LVESV at six-months of follow-up compared to baseline.

### Healthy control group

The healthy control group (HG) was constituted by 11 sex- and age-matched healthy individuals. Defined inclusion criteria were normal lipid profile (including cholesterol (< 240 mg/dL), high-density lipoprotein (HDL) cholesterol (> 60 mg/dL), low-density lipoprotein (LDL) cholesterol (between 130 to 159 mg/dL) and triglycerides (< 150 mg/dL), normal body mass (body mass index (BMI) between 18.5 to 24.9 range), and normal cardiac evaluation. Exclusion criteria were family history of heart disease and/or cardiomyopathy; active infections or inflammatory process; autoimmune, neoplastic, and allergic diseases; use of any drug within 30 days before inclusion and inability to understand informed consent.

### Blood samples

At admission, just before the device implantation, peripheral blood (PB) samples were taken in all patients to determine haematological parameters and chemistry assessment (including glycemia, creatinine, high sensitivity C-reactive protein (hsCRP), brain natriuretic peptide (BNP) and uric acid. In addition, to perform the analysis of inflammatory parameters, PB samples from each patient at T0 and T6 and from healthy individuals were collected into K3-EDTA, heparin and serum tubes.

### Quantification of T cells subpopulations

Quantification of total T cells (CD3^+^), and Th (CD4^+^) and Tc (CD8^+^) subsets was performed using Lymphogram reagent (Cytognos, Salamanca, Spain), consisting of a mix of monoclonal antibodies (mAb): anti-CD19 and anti-CD8 conjugated with fluorescein isothiocyanate (FITC), anti-CD56 and anti-CD3 conjugated with phycoerythrin (PE), and anti-CD4 conjugated with Cyanine 5 tandem (PECy5). As described by others [42], Lymphogram reagent (Cytognos, Salamanca, Spain) was added to aliquots from PB sample, collected in K_3_-EDTA, and incubated for 15 min at room temperature in darkness. After incubation period, lyse and wash protocol was followed: 2 ml of FACS Lysing Solution (BDB, San Jose, CA) (previously diluted 1:10 (vol/vol) in distilled water) were added to each sample and, after 10 min of incubation, cells were washed with 2 mL of phosphate buffer saline (PBS). In the end, cells were resuspended in 0.5 mL of PBS and acquired in FACSCalibur flow cytometer (BD).

### Quantification of peripheral regulatory T cells

The immunofluorescent staining of Treg cells was performed according to a protocol established by others [42–44]. Aliquots for Treg evaluation were made from a PB sample, collected in K3-EDTA, and anti-CD25-FITC (clone M-A251; Pharmingen BD, San Jose, CA, USA), anti-CD127-PE (clone hIL-7R-M21; Pharmingen BD, San Jose, CA, USA), and anti-CD4-peridinin-chlorophyll proteins-cyanine 5.5 (PerCP-Cy5.5) (clone SK3; Pharmingen BD, San Jose, CA, USA) were added. The aliquots were incubated for 15 min at room temperature in darkness, followed by the lyse and wash protocol described above.

### Immunophenotypic and functional characterization of Th and Tc cells

PB T cells, collected in a heparin tube, were submitted to in vitro stimulation with PMA/ ionomycin, in the presence of brefeldin A, according to the immunofluorescence staining protocol described by others [42, 43]. Briefly, 500 µL of each PB sample were diluted 1:1 (vol/vol), in RPMI-1640 medium (Gibco, Life Technologies, Paisley, Scotland, UK), supplemented with 2 mM L-glutamine. T cells were stimulated with 50 ng/mL of phorbol 12-myristate 13-acetate (PMA) (Sigma, Saint Louis, MO, USA), 1 µg/mL of ionomycin (Sigma) and 10 µg/mL of Brefeldin A (Sigma). The samples were incubated for 4 h at 37ºC, in a humidified incubator with 5% CO_2_ concentration.

Each cultured PB sample was aliquoted in three different tubes (200 mL/tube) and incubated with the following monoclonal antibodies: anti-CD4-PerCP (clone SK3; Becton Dickinson Biosciences (BD), San Jose, CA, USA) and anti-CD8-allophycocyanin (APC) (clone B9.11; Beckman Coulter – Immunotech, Marseille, France). In order to analyse the intracellular expression of interleukin (IL)-2, tumour necrosis factor (TNF)-α, and interferon (IFN)-γ by Th and Tc cells, cell permeabilization protocol, using IntraPrep Permeabilization Reagent (Beckman Coulter, Brea, CA, USA), and intracytoplasmatic staining protocol were followed, according to manufacturer's instructions. All cell aliquots were stained separately with IL-2 (clone MQ1-17H12; BD Pharmingen, San Diego, CA, USA), TNF-α (clone MAb11; BD Pharmingen), and interferon (IFN)-γ (clone 4S.B3; BD Pharmingen), all conjugated with FITC. Finally, cells were resuspended in 0.5 mL of PBS (Gibco BRL, Life Technologies, Vienna, Austria) and then acquired in a FACSCalibur flow cytometer (BD).

### Flow cytometry data acquisition and analysis

FACS analysis was performed blinded for patient’s clinical information.

FACSCalibur flow cytometer (BD) was used to perform flow cytometry data acquisition.

The percentage of positive cells within each cell subset and/or their mean fluorescent intensity (MFI) were measured.

**Fig. 1:**
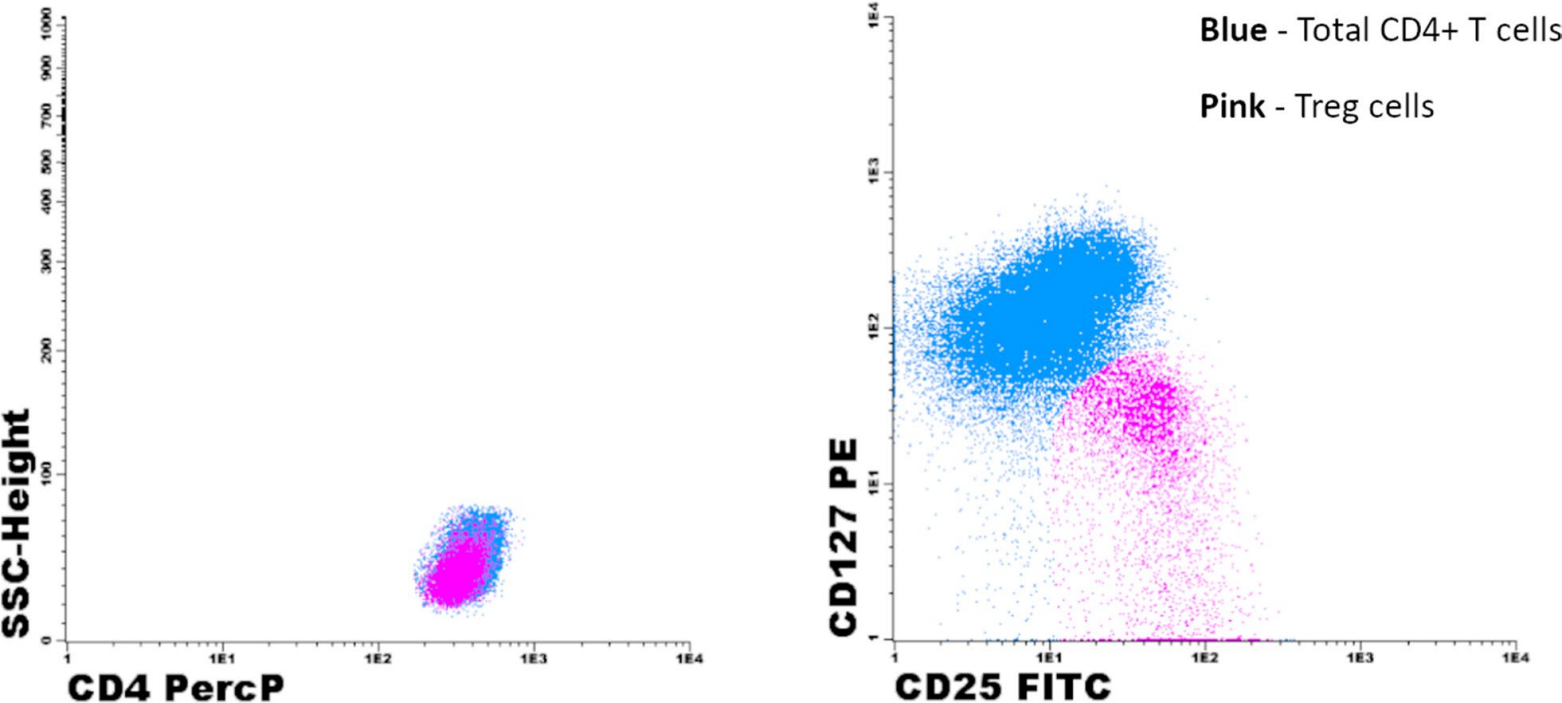
Representative dot plots illustrating the identification of Treg cells in peripheral blood samples using a combination of anti-CD25-FITC, anti-CD127-PE and anti-CD4 PerCP-Cy5.5: CD4^+^/CD127Low/CD25High

The identification and quantification of Treg were made according to the expression of the following phenotype: CD4^+^/CD25^bright^/CD127^−/low^, after a first acquisition of 20 000 total events, followed by an acquisition on an electronic CD4^+^ gate. The strategy used for quantification of peripheral Treg cells is represented in Fig. 1.

For immunophenotypic and functional characterization of Th and Tc cells, T lymphocytes were identified according to their typical light scatter. Cytokine production was evaluated in both Th (CD4^+^) and Tc (CD8^+^) cells on an electronic gate with at least 20.000 events, after a first acquisition step of 20 000 total events.

Data were analysed using the Infinicyt™ software, V.1.5 (Cytognos SL, Salamanca, Spain) and absolute counts were determined using two different instrumentation platforms (flow cytometer and haematological cell analyser).

#### Statistical analysis

Statistical analysis was performed using the non-parametric Mann–Whitney U test for independent variables. The Wilcoxon signed-rank test was used to compare T0 vs. T6 [45]. Results were expressed as mean ± standard deviation or median (range). All statistical analyses were performed using R Core Team (2017). R: A language and environment for statistical computing. R Foundation for Statistical Computing, Vienna, Austria. URL http://www.R-project.org/), (version 3.4.1). Differences were considered to be statistically significant when p value was < 0.05.

Prespecified analysis plan is detailed in Additional file 1: Table S1.

## Results

### Baseline characteristics of Healthy Control Group

HG was constituted by eight males and three females with an average age of 43.4 ± 10.8 years old. Considering the lipid profile, HG showed the following mean values: cholesterol: 184.3 ± 16.1 mg/dL; HDL cholesterol: 64 ± 8.6 mg/dL; LDL cholesterol: 96.5 ± 11.4 mg/dL and triglycerides: 119.1 ± 24.0 mg/dL. Comparing to HF patients, HG presented a significantly higher value of HDL Cholesterol (*P* < 0.001). BMI average of HG was 22.8 ± 1.3.

### Clinical characteristics of responders and non-responders to CRT

The characteristics of the global HF population are described in Table 1.

Regarding chronic medication, 72.2% of the patients were under ACE inhibitors, 19.4% under angiotensin II type 1-receptor blockers, 94.4% under beta-adrenergic blockers, 66.7% under spironolactone, 97.2% under furosemide, 27.8% under digoxin, 50% under statins, and 13.9% under ivabradine, before CRT.

Before CRT, the majority of patients were in NYHA class III or IV (79.5%). At the six-month follow-up, the proportion of responders to CRT was 54%, according to the echocardiographic definition. There were no changes in medication between baseline and the six-month re-evaluation.

Regarding baseline characteristics, responders to CRT were significantly older than non-responders and presented higher levels of HDL cholesterol. We found no other statistically significant differences in clinical characteristics. As expected, after CRT, responders presented significantly lower BNP levels compared to non-responders to CRT and significantly better LV geometry and systolic function (Table 1).

### Evaluation of T Cells subpopulations in heart failure patients by comparison with healthy group

Considering the subpopulations of T cells, no significant differences were found in the absolute numbers of Th and Tc cells between HG and HF patients (Table 2).

**Table 2:** Comparative analysis of overall T cells and their subsets in healthy individuals and patient groups

		HG (n = 11)	HG vs HFP-T0 *P* values	HFP (n = 39)		HG vs HFP-T6 *P* values
				HFP-T0	HFP-T6	
*WBC*	10^3^/ µl	7.52 ± 2.07	0.170	8.36 ± 1.71	8.34 ± 1.76	0.202
*T cells*	%	76.10 ± 9.50	0.513	77.39 ± 9.95	77.07 ± 10.73	0.699
	Cells/µl	1510.45 ± 381.77	0.717	1467.14 ± 638.21	1481.90 ± 588.58	0.885
*Th cells*	%	66.22 ± 7.87	0.545	63.63 ± 10.85	61.99 ± 12.91	0.326
	Cells/µl	994.93 ± 258.20	0.331	919.55 ± 426.25	918.63 ± 383.68	0.449
*Tc cells*	%	28.78 ± 7.35	0.578	31.33 ± 10.84	33.67 ± 12.27	0.302
	Cells/µl	439.63 ± 174.87	1.000	474.22 ± 274.04	519.76 ± 293.35	0.647

Results are expressed as mean ± standard deviation

Statistically significant differences were considered when *P* < 0.05 (Mann–Whitney U test and Wilcoxon signed-rank)

*Th* T helper (CD4^+^) cells, *Tc* T cytotoxic (CD8^+^) cells, *HG* healthy control group, *HFP* heart failure patients, *HFP-T0 (T0)* baseline assessment, *HFP-T6 (T6)* follow-up evaluation, 6 months after Cardiac Resynchronization Therapy (CRT), *WBC* white blood cells

**Fig. 2:**
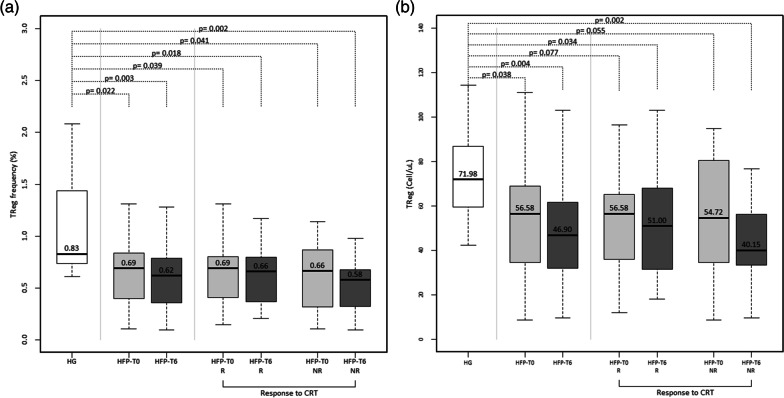
Frequency (%) (**a**) and absolute number (Cell/μl) (**b**) of peripheral regulatory CD25^bright^/CD127^low^ CD4^+^ T cells in total leukocytes, from healthy individuals (HG) and heart failure patients, at baseline assessment (HFP-T0) and 6 months after cardiac resynchronization therapy implantation (HFP-T6). Heart failure patients were distributed: according to response to cardiac resynchronization therapy: responders (R) and non-responders (NR). Statistically significant differences were considered when *P* < 0.05

However, regarding Treg cells, HF patients showed significantly lower frequency and absolute values of these cells at baseline and even six months after CRT compared with healthy individuals (Fig. 2a, b).

### Impact of CRT on T cells subpopulations and differences according to CRT response

As shown in Table 3, comparing responders to non-responders to CRT, we found that non-responders presented a significantly higher frequency of total T cells at baseline and this difference remained after CRT. However, regarding the subpopulations Th and Tc cells, no significant differences were observed between responders and non-responders to CRT, neither for the baseline values, nor for the six-month follow-up quantification (Table 3). Likewise, when comparing baseline (T0) and follow-up (T6) assessments, no significant differences were also found in HF patients.

**Table 3:** Comparative analysis of overall T cells and their subsets in patient groups, according to response to CRT

		HFP (n = 39)		HFP-T0 vs HFP-T6 *P* values	Responders (n = 21)		HFP-T0 vs HFP-T6 (R) *P* values	Non-Responders (n = 18)		HFP-T0 vs HFP-T6 (NR) *P* values	R vs NR (HFP-T0) *P* values	R vs NR (HFP-T6) *P* values
		HFP-T0	HFP-T6		HFP-T0	HFP-T6		HFP-T0	HFP-T6			
*According to response to CRT*												
*WBC*	10^3^/ µl	8.36 ± 1.71	8.34 ± 1.76	0.726	8.10 ± 1.58	8.10 ± 1.50	0.672	8.66 ± 1.84	8.64 ± 2.06	0.842	0.242	0.220
*T cells*	%	77.39 ± 9.95	77.07 ± 10.73	0.633	**73.53 ± 11.44**	**73.33 ± 10.97**	0.225	**81.47 ± 6.04**	**81.99 ± 8.38**	0.433	**0.026**	**0.015**
	Cells/µl	1467.14 ± 638.21	1481.90 ± 588.58	0.326	1412.74 ± 573.74	1470.27 ± 611.70	0.768	1524.56 ± 712.17	1497.15 ± 576.25	0.404	0.518	0.892
*Th cells*	%	63.63 ± 10.85	61.99 ± 12.91	0.736	65.71 ± 10.77	62.76 ± 12.50	0.523	61.43 ± 10.78	61.02 ± 13.75	0.900	0.159	0.404
	Cells/µl	919.55 ± 426.25	918.63 ± 383.68	0.589	900.76 ± 324.14	930.13 ± 389.04	1.000	939.37 ± 522.19	904.26 ± 389.07	0.495	0.940	0.718
*Tc cells*	%	31.33 ± 10.84	33.67 ± 12.27	0.270	29.92 ± 10.21	33.11 ± 12.54	0.580	32.82 ± 11.57	34.37 ± 12.28	0.501	0.391	0.560
	Cells/µl	474.22 ± 274.04	519.76 ± 293.35	0.191	449.96 ± 284.46	515.26 ± 301.82	0.832	499.83 ± 268.33	525.40 ± 292.15	0.211	0.425	0.888

Bold: Statistically significant differences (*P* < 0.05)

Results are expressed as mean ± standard deviation

Statistically significant differences were considered when *P* < 0.05 (Mann–Whitney U test and Wilcoxon signed-rank)

*Th* T helper (CD4^+^) cells, *Tc* T cytotoxic (CD8^+^) cells, *HFP* heart failure patients, *HFP-T0 (T0)* baseline assessment, *HFP-T6 (T6)* follow-up evaluation, 6 months after Cardiac Resynchronization Therapy (CRT), *R* responders, *NR* non-responders, *WBC* white blood cells

Analysing the impact of CRT in Tregs frequency, we found that both responder and non-responder patients displayed lower frequency and absolute values of Treg compared to HG, in both moments of the evaluation (Fig. 2a, b).

### Functional characterization of peripheral blood Th and Tc cells of heart failure patients

**Fig. 3:**
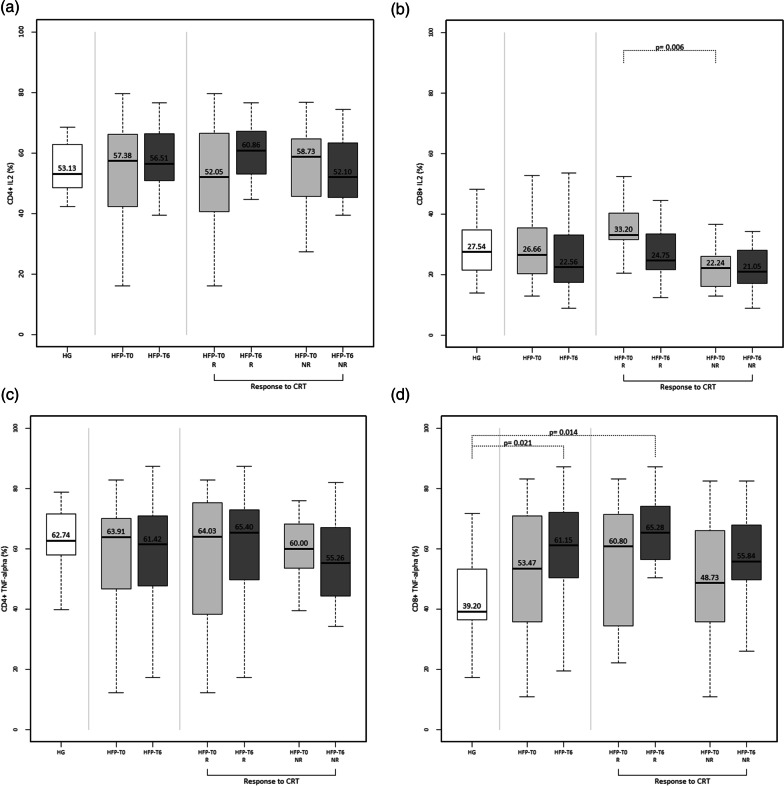

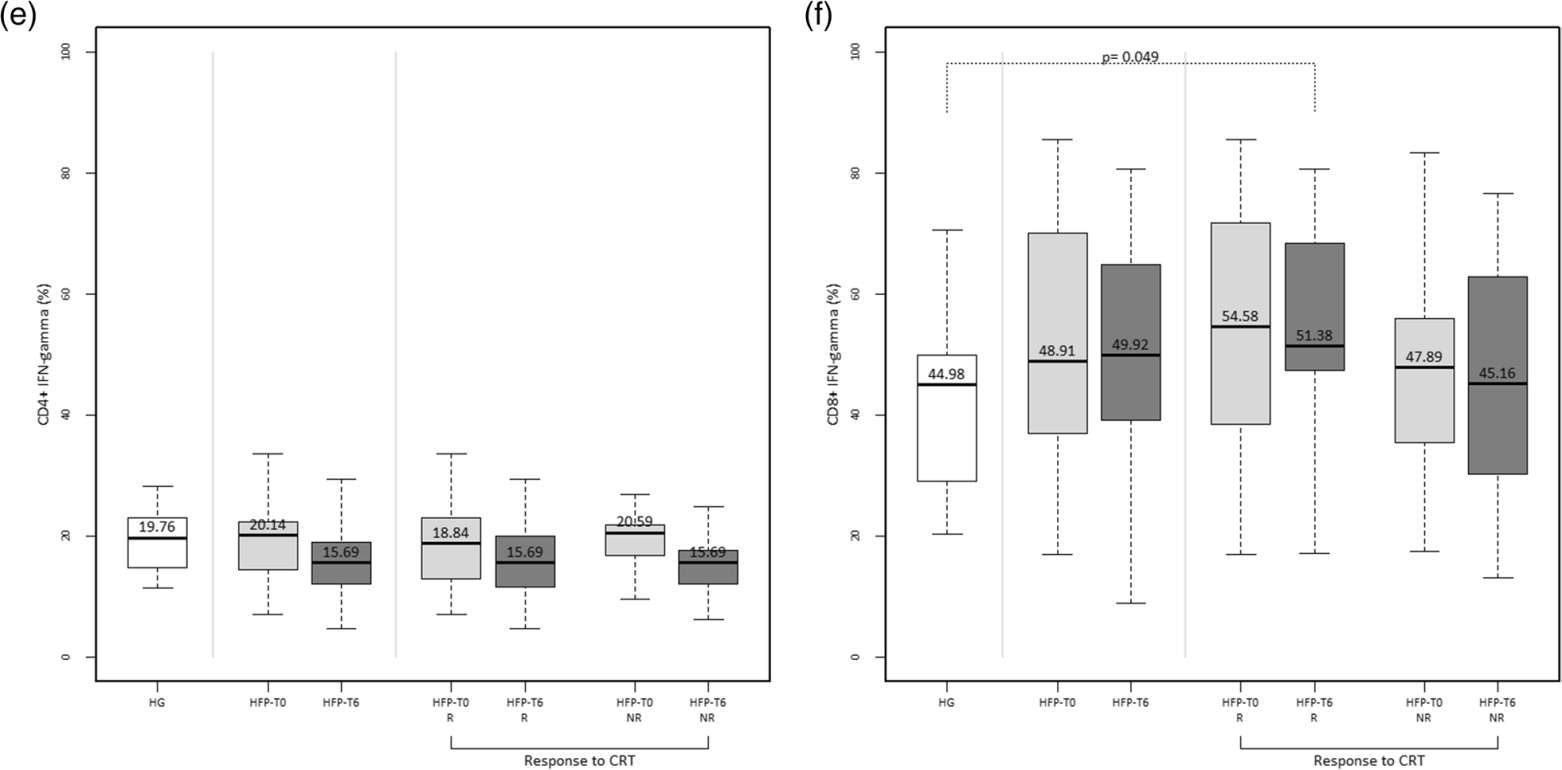
Functional characterization of peripheral T helper (CD4^+^) cells and T cytotoxic (CD8^+^) cells. The percentage of T helper cells producing IL-2 (**a**), TNF-α (**c**) and IFN-γ (**e**) and T cytotoxic cells producing IL-2 (**b**), TNF-α (**d**) and IFN-γ (f) were evaluated in healthy individuals (HG) and heart failure patients at baseline assessment (HFP-T0) and 6 months after cardiac resynchronization therapy implantation (HFP-T6). Heart failure patients were divided according to response to cardiac resynchronization therapy: responders (R) and non-responders (NR). Statistically significant differences were considered when *P* < 0.05

Regarding the frequency of Th cells producing IL-2 (Fig. 3a), TNF-α (Fig. 3c) and IFN-γ (Fig. 3e), there were no significant differences between the overall HF patient´s population and the HG. Moreover, no significant differences were observed when comparing HF patients at baseline with post-CRT (T0 and T6). According to CRT response, we also found no significant differences in the frequency of these T cells between responders and non-responders.

Considering Tc cells producing IL-2, no significant differences were found between HF patients and healthy individuals (HG Vs HFP-T0: *P* = 0.903 and HG Vs HFP-T6: *P* = 0.429) (Fig. 3b). However, responders to CRT presented, a significantly higher frequency of IL-2 producing Tc cells at baseline than non-responders (Fig. 3b).

Another difference was observed in the frequency of TNF-α producing Tc cells, which was significantly higher in HF patients six-months post-CRT compared with healthy individuals (HG Vs HFP-T0: *P* = 0.336 and HG Vs HFP-T6: *P* = 0.021) (Fig. 3d). This difference was even more evident in responders to CRT (Fig. 3d).

Moreover, responders to CRT also presented a significantly higher percentage of Tc cells expressing IFN-γ cells after CRT, compared to the control group (Fig. 3f).

No other differences were seen between T0 and T6 nor between responders and non-responders concerning TNF-α and IFN-γ producing Tc cells (Fig. 3d and f).

## Discussion

The main findings of the present work can be summarized as follows: (1) Treg cells are decreased in CHF patients and CRT seems not to be able to restore their normal levels; (2) responders to CRT presented a higher frequency of Tc cells producing IL-2 at baseline than non-responders and a higher percentage of Tc cells expressing TNF-α and IFN-γ after CRT than healthy individuals. (3) CHF patients showed similar levels of overall T cells and Th and Tc subsets to those observed in healthy controls.

Treg cells are major mediators of immune tolerance [46] and homeostasis, preventing auto-immune diseases and controlling inflammation [46, 47]. They can act suppressively in innate and adaptive immune response in a direct way: triggering a direct cellular action with the secretion of cytokines such as IL-10, TGF-β and IL-35; and indirectly: expressing high levels of CD25, competing with effector T cells for IL-2, and thus limiting their proliferation [48].

In the failing heart, Treg cells play a cardioprotective role, regardless of HF aetiology [49]. In animal myocardial ischaemia/reperfusion injury models, Treg cells are presented as responsible for attenuating cardiomyocyte apoptosis and activating a pro-survival pathway [50]. In virus-induced myocarditis cases, Tregs cells also suppress immunopathology and prevent tissue damage, avoiding the progression of the disease [51]. Another example is found in myocardial infarction mice studies, where therapeutic Tregs activation increases de novo collagen expression within the scar [52, 53]. Beyond that, Treg activation attenuates interstitial fibrosis, myocardial matrix metalloproteinase activity and cardiac apoptosis, and decreases neutrophils, macrophages, and lymphocytes infiltration as well as TNF-α and IL-1β production [5]. Conversely, Tregs depletion in infarcted mice accelerates ventricular dilation and accentuates apical remodeling [54].

In clinical studies, the frequency of circulating Treg cells is decreased in patients with CHF which may contribute to disturbed immune regulation, chronic inflammation [9, 55] and progression of HF [13, 41]. A study performed by Tang TT et al. (2010) demonstrated that patients with CHF presented not only a decrease in Treg cells numbers but also a loss of suppressive capacity on proliferation and production of cytokines. They also described an inverse correlation between the suppressive function of Treg cells and the severity of the disease, suggesting that reduced levels of these cells may be responsible for uncontrolled T cell activation and consequently for myocardial injury and aggravation of cardiac function [55].

According to these studies, our results confirm that circulating Treg cells are decreased in CHF patients. On the other hand, Tc cells producing TNF-α are significantly increased, indicating that there is an alteration in T-cell homeostasis in CHF patients. We can assume that the increased frequency of pro-inflammatory cytokines-producing T cells may be related to the decreased frequency of Treg cells.

According to their cytokine secretion, effector T cells can be divided into several subpopulations. As known, Th type 1 cells express IFN-γ (its signature cytokine) [12, 56–58], TNF-α and IL-2 [12, 57] whereas Th2 produce high amounts of IL-4 and IL-5 [56, 58]. More recently, the existence of IL-17-producing Th cells, designated Th17, were also described as the third subset of CD4.^+^ effector T cells [56, 57, 59].

Th1 and Tc1 cells are potent effector cells through the secretion of IFN-γ and TNF-α. However, type 1 immunity might also play a pathogenic role in several pathologies, including autoimmune disorders, and chronic inflammatory disorders [57]. In CHF, immune activation can be initiated by direct antigenic stimulation or secondary to cardiac injury, with exposure of "new antigens" that consequently trigger an immune response against the heart. In either case, the immune pathways that follows are similar and implicate the development of T cell–specific responses [6, 60], as well as antibody responses and complement activation [60].

Prior studies in CHF patients established a shift in Th1/Th2 balance towards Th1 and a shift in Th17/Treg balance towards Th17 [9, 12, 55]. Furthermore, the increased Th1 response in HF is proportional to the severity of the disease [10]. However, little is known about the role of human Tc cells in CHF.

In the present study, we found a higher baseline percentage of Tc cells expressing IL-2 in responders compared to non-responders. Similarly, patients with HF, especially responders, presented an increased frequency of TNF-α and IFN-γ producing Tc cells compared to the control group. These results suggest that the type 1 Tc cells phenotype may be important to reverse remodeling and CRT response. No differences in the percentage of Tc cells expressing TNF-α and IFN-γ were found between healthy controls and CHF patients at baseline, as well as between responders and non-responders.

Patients responding to CRT have shown a reduction of inflammatory status in small and medium-sized clinical studies [61–64]. In fact, Michelucci et al. (2007), observed a decrease in IL-6 and high sensitivity C-reactive protein (hsCRP) in 140 HF patients who underwent an evident reverse remodeling with CRT [62]. Moreover, Lappegård et al. (2006), in a small clinical study with 9 HF patients, also found a reduction of inflammatory parameters such IL-6, IL-8 and monocyte chemoattractant protein-1 (MCP-1) after CRT [63]. Additionally, a recent study carried out by Gambardella J et al. (2021) showed that baseline level of glycation of type 1 ryanodine receptor in circulating lymphocytes can be used as a novel independent biomarker of CRT response [65].

However, no changes in serum markers or inflammatory mediators were reported in other studies [64, 66, 67]. Boriani et al. [66] and Tarquini et al. [67] showed that CRT had no effect on inflammatory markers such as IL-6, TNF, and soluble TNF receptors. Within these unclear settings, doubts about the impact of CRT on inflammatory mediators’ neutralization may arise, despite its beneficial effects on symptoms and cardiac remodeling.

After myocardial injury, T effector cells promote apoptosis of cardiomyocytes [49] In animal models, it is also well established that Th1 and Th17 cells can induce cardiac fibrosis and adverse cardiac remodeling [11, 68, 69]. On the other hand, Tregs protect LV remodeling and induce an anti-inflammatory milieu by inhibition of neutrophils, monocytes and T cells accumulation and consequent inflammatory cytokine production (such as TNF-α, IL-1β, IFN-γ) [49]. However, little is known about the impact of CRT on the inflammatory response mediated by T cells. To the best of our knowledge this is the first study that has evaluated the impact of CRT on circulating Th1/Tc1 cells and Treg cells.

Here we suggest that CRT had no impact on the frequency and absolute values of Treg cells in patients with HF. Despite the decreased Treg cells values, CRT does not appear to have a therapeutic ability to correct Treg cells levels to normal, even in responders. In the same way, regarding pro-inflammatory cytokine producing Tc1 cells, no differences were found in CHF patients from baseline assessment to follow-up. Moreover, it was after CRT that we found the greatest difference in TNF-a and IFN-g producing Tc cells between control and patient groups. These observations suggest that CRT does not decrease the frequency of pro-inflammatory cytokine producing Tc1 cells even in responder patients. Nevertheless, the frequency of IL2-producing Tc cells in responders seems to stabilize to similar values to those of the control group, after CRT.

Taken together, considering the pattern of chronicity and natural course of the CHF, our data supports the idea that inflammation mediated by T cells continues to expand despite CRT even in responders (regardless of reverse remodeling).

## Conclusion

T cell subpopulations are altered in patients with HF, which may result in an exacerbated pro-inflammatory pathway (Treg cells decline *plus* predominance of a Tc1 cells phenotype). Our results suggest that CRT cannot restore Treg cells or inhibit T cell-mediated pro-inflammatory pathway. It seems that the inflammatory condition underlying HF continues to evolve with the progression of the disease despite CRT.

## Limitations

Healthy controls were selected according to clinical history, considering available and recent analytical results and cardiac exams. But the inclusion and exclusion criteria were not extensively evaluated as for the patient group. The control group was constituted by healthy and active people who apparently did not have comorbidities such as those presented in the exclusion criteria for HF patients. However, we did not parentally exclude these same morbidities.

Another important limitation of our study was a small sample size. Especially when comparing patients with the control group or comparing responders and non-responders, statistical power could be partially lost. Our work included multiple comparisons between groups with a smaller sample size that may not be representative of the population under study due to random sampling error. Further studies with larger number of samples are required to evaluate whether the alleviation or worsening of inflammatory status mediated by T cells translates into different prognosis after CRT.

## Supplementary Information

**Additional file 1: Supplementary Table 1.** Prespecified analysis plan.

## Data Availability

The main data were obtained at FACSCalibur flow cytometer and were presented within the article. For statistical analysis, and graphical construction, *.xlsx*, *.txt* and *.r* formats were used. These derived data supporting the results are available on reasonable request from the corresponding author, Paiva A. Data are not publicly available due to the presence of personal identification of research participants, which could compromise privacy rights.
