# Supplementary material for: Reduced numbers of regulatory T cells in chronic heart failure seems not to be restored by cardiac resynchronization therapy
Source: BMC Cardiovasc Disord. 2023 Feb 15;23:89. doi: 10.1186/s12872-023-03109-x (PMC9933267; doi:10.1186/s12872-023-03109-x)
Supplement: Supplementary file 1 — Additional file 1: Supplementary Table 1. Prespecified analysis plan. [file 12872_2023_3109_MOESM1_ESM.docx]

**Supplementary Table 1** – Prespecified analysis plan.

| **Main hypothesis** | | |
| --- | --- | --- |
| CRT can have an impact on the T cell-mediated inflammatory response. | | |
| **Prespecified hypotheses** | **Group comparison** | **Evaluated parameters** |
| Patients have altered numbers and functional activity of T cells. | **HF patients**  *versus*  **Control group** | Frequency and absolute number of T CD3^+^, TCD4^+^ (Th), TCD8^+^ (Tc) and Treg cells, and frequency of Th and Tc cells producing pro-inflammatory cytokines (IL-2, TNF-α, IFN-γ) |
| CRT can reduce the functional activity of T cells and restore Treg cell numbers. | **HF patients at T0**  (before CRT)  *versus*  **HF patients at T6**  (6 months follow-up post-CRT) |  |
| Responders to CRT present, at baseline, lower inflammatory values compared to than non-responders. | **Responders**  *versus*  **Non-responders** |  |
